# *SLC52A2*-Related Riboflavin Transporter Deficiency in Non-Isolated Auditory Neuropathy: Evidence from a Chinese Cohort and a Global Variant Review

**DOI:** 10.3390/genes17101260

**Published:** 2026-10-11

**Authors:** Li Wang, Jin Li, Wenjing Wang, Mengtao Song, Yi Luo, Hongyang Wang, Qiuju Wang

**Affiliations:** 1 School of Medicine, Nankai University, Tianjin 300071, China; wanglizonly@mail.nankai.edu.cn; 2 Senior Department of Otolaryngology Head and Neck Surgery, The 6th Medical Center of Chinese PLA General Hospital, Chinese PLA Medical School, Beijing 100853, China; lijin199505@163.com (J.L.); wenjingwang12@163.com (W.W.); song_mengtao@163.com (M.S.); luoyi301ent@163.com (Y.L.); 3 State Key Laboratory of Hearing and Balance Science, Beijing 100853, China

**Keywords:** *SLC52A2*, riboflavin transporter deficiency type 2, auditory neuropathy, genotype–phenotype correlation, variant spectrum, riboflavin

## Abstract

**Background/Objectives**: Riboflavin transporter deficiency type 2 (RTD2), caused by biallelic pathogenic variants in *SLC52A2*, is a treatable neurodegenerative disorder in which auditory neuropathy may be an early manifestation. This study characterized the clinical and genetic spectrum of *SLC52A2*-related RTD2, with emphasis on auditory findings and geographic variation in the reported variant spectrum. **Methods**: We screened unrelated probands with clinically diagnosed auditory neuropathy from a multicenter cohort for *SLC52A2*-related disease. Clinical, audiological, genetic, treatment, and follow-up data were reviewed. PubMed, Web of Science, and Embase were searched from inception through Feb 2026 to summarize reported *SLC52A2* variants. **Results**: Four patients carried eight distinct *SLC52A2* variants, four of which were previously unreported. Audiological testing identified an auditory neuropathy phenotype, with onset between 0.5 and 4 years of age and severity ranging from mild or moderate to profound. In two patients, hearing loss was the first or a concurrent initial manifestation. Riboflavin treatment improved anemia, ataxia, muscle strength, and motor function, whereas established auditory and visual impairment showed limited recovery. The Chinese cohort showed a dispersed variant distribution across *SLC52A2*, without clustering at known hotspots. In the global dataset, p.G306R was the most frequent variant, accounting for 41.6% (74/178) of reported variant alleles. **Conclusions**: *SLC52A2*-related RTD2 should be considered in children with early-onset or progressive auditory neuropathy and multisystem manifestations. Early molecular diagnosis may permit timely riboflavin treatment before irreversible neural injury develops. The four previously unreported variants expand the *SLC52A2* variant spectrum, while the geographic patterns identified in published cases require validation in larger cohorts.

## 1. Introduction

Riboflavin transporter deficiencies (RTDs) are congenital metabolic disorders caused by pathogenic variants in the SLC52 gene family, including *SLC52A1* (solute carrier family 52 member 1), *SLC52A2* (solute carrier family 52 member 2) and *SLC52A3* (solute carrier family 52 member 3) [1]. *SLC52A2*-related RTD2 is associated with the OMIM phenotype Brown-Vialetto-Van Laere syndrome 2 (BVVLS2; MIM #614707). RTD2, which is caused by biallelic pathogenic *SLC52A2* variants, is the most common subtype, although fewer than 200 cases have been reported worldwide [2]. Its estimated prevalence is below one per million [3]. Given its rarity, the true prevalence may be underestimated because some patients remain undiagnosed or misdiagnosed [4]. *SLC52A2* is located at 8q24.3 and encodes riboflavin transporter 2 (RFVT2) [5], which mediates transmembrane riboflavin transport [6]. Impaired *SLC52A2* function may disrupt energy metabolism in the nervous system and contribute to a progressive neurodegenerative phenotype [7]. The clinical manifestations of RTD2 are highly heterogeneous and may include hearing loss, sensory ataxia, muscle weakness, optic atrophy, nystagmus, bulbar dysfunction, respiratory insufficiency, and hematological abnormalities, including anemia [8].

Among these multisystem manifestations, sensorineural hearing loss is one of the most characteristic clinical features of RTD2 [9,10,11,12,13]. This may be because the auditory system is particularly vulnerable to disruptions in energy metabolism. Bilateral sensorineural hearing loss occurs in 83.9% of patients during the disease course [14]. In the early stages, outer hair cell function may be preserved despite impaired auditory neural conduction, indicating a predominantly retrocochlear lesion [15,16]. Previous studies have suggested that RTD2-related hearing loss is predominantly auditory neuropathy [12,17,18], likely associated with degeneration of the vestibulocochlear nerve [19,20]. In the context of multisystem involvement, RTD2 therefore represents a form of non-isolated or syndromic auditory neuropathy [21].

Recognition of this auditory presentation is clinically relevant because RTDs may progress rapidly. The mean interval from first symptom to death has been reported to be less than 5 years [22], expected survival in insufficiently treated patients is less than 12 years [4], and the median survival time is 7.5 years [23]. Because RTD2 is rare and clinically heterogeneous, diagnosis remains challenging and is often delayed. The overall diagnostic delay has been estimated at 5 to 6 years [14]. In some patients, hearing loss may be the initial or only early manifestation and may precede clinically evident motor neuron involvement [24]. Systematic characterization from an audiological perspective may therefore facilitate earlier recognition of RTD2 and timely initiation of treatment.

In this study, we identified four Chinese patients with the rare disorder *SLC52A2*-related RTD2 from a multicenter auditory neuropathy cohort. We examined the temporal relationship between hearing loss and extra-auditory manifestations, and assessed which clinical features responded to riboflavin treatment. We also reviewed reported *SLC52A2*-related cases and compared variant types, hotspot variants and geographic distribution to inform genetic testing and counseling.

## 2. Materials and Methods

**Figure 1:**
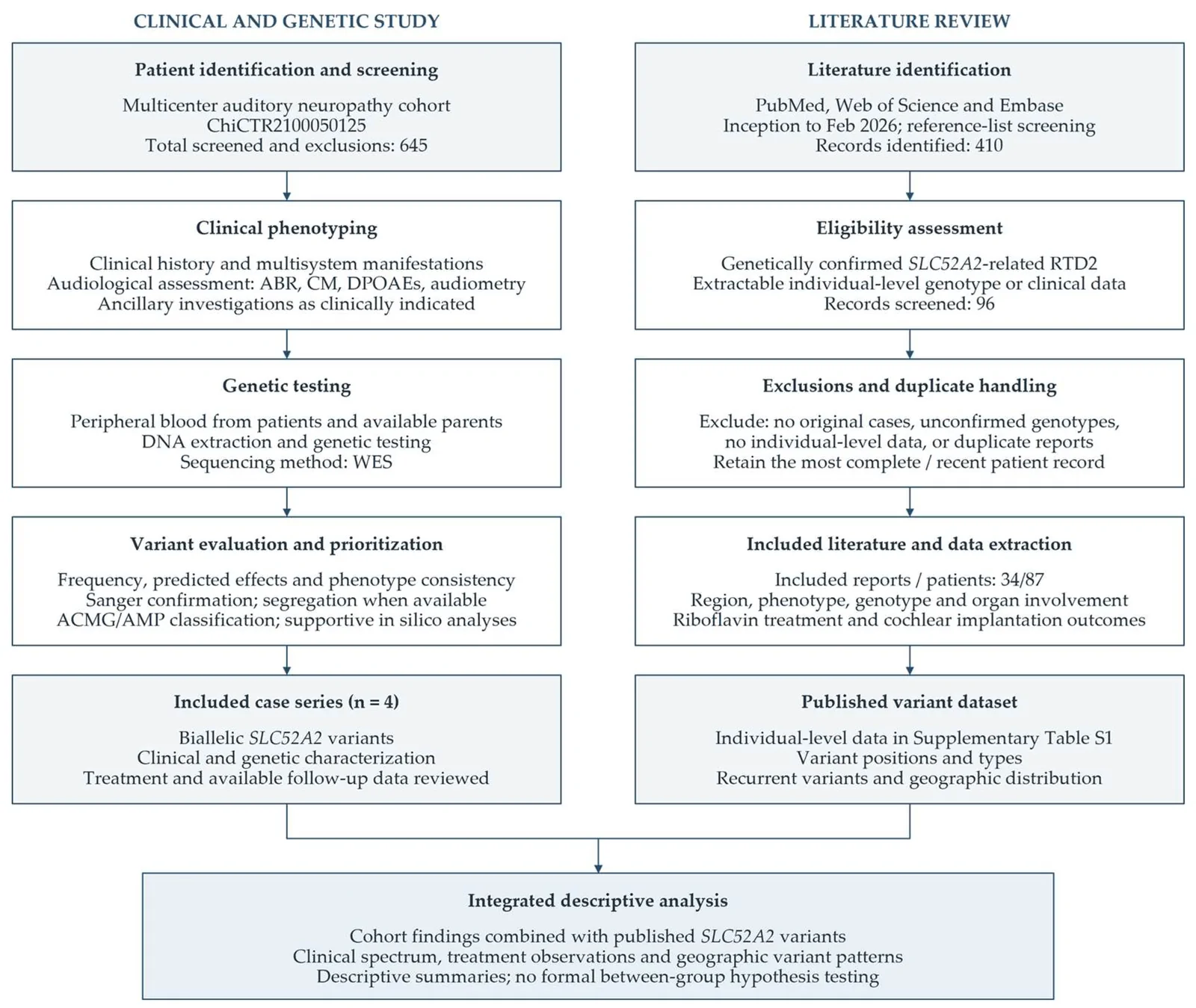
Patients were identified from a multicenter auditory neuropathy cohort, and their clinical, audiological, genetic, and available treatment and follow-up data were reviewed. Candidate *SLC52A2* variants were evaluated using annotation, Sanger confirmation, familial segregation where available, and ACMG/AMP classification. In parallel, a literature search identified published cases with extractable individual-level data. After eligibility assessment and removal of duplicate reports, published variants were integrated with those identified in this cohort for descriptive analysis of the variant spectrum and geographic distribution. ABR, auditory brainstem response; ACMG/AMP, American College of Medical Genetics and Genomics/Association for Molecular Pathology; CM, cochlear microphonic; DPOAEs, distortion product otoacoustic emissions; RTD2, riboflavin transporter deficiency type 2; WES, whole-exome sequencing.

The overall study workflow, including patient identification, clinical phenotyping, genetic analysis, and literature review, is summarized in Figure 1.

### 2.1. Study Participants and Inclusion Process

This retrospective clinical and genetic study screened 645 patients enrolled in the multicenter study Clinical Diagnosis and Intervention of Acoustic Neuropathy (ChiCTR2100050125) [25]. Auditory neuropathy was diagnosed based on absent or clearly abnormal ABR with preserved CM responses and/or DPOAEs [26,27,28]. Patients underwent clinical phenotyping and genetic testing by whole-exome sequencing. Four patients with biallelic *SLC52A2* variants were identified and included for clinical, genetic, treatment, and follow-up analyses. The study was approved by the Committee of Medical Ethics of Chinese PLA General Hospital (S2020-228-01; 20 August 2020).

### 2.2. Clinical Audiological and Ancillary Evaluations

Clinical records and follow-up interviews were reviewed to collect data on sex, current age, age at onset, first or early manifestations, perinatal history, neurological manifestations, ocular involvement, hematologic abnormalities, respiratory involvement, treatment history and follow-up outcomes. Available audiological evaluations included pure-tone audiometry or behavioral audiometry, ABR, DPOAEs, CM, and acoustic immittance. Electrocochleography and speech recognition testing were performed when clinically appropriate. Auditory neuropathy was defined as abnormal or absent ABR with preserved CM and/or DPOAEs. Additional investigations, including cranial or internal auditory canal magnetic resonance imaging (MRI), electroencephalography, electromyography, visual evoked potentials, and other systemic examinations, were performed as clinically indicated.

### 2.3. Genetic Testing and Variant Interpretation

Peripheral blood samples were obtained from the probands and available parents, and genomic DNA was extracted for whole-exome sequencing (WES). Exome capture and next-generation sequencing were performed using laboratory-specific protocols. Sequence data were analyzed against the human reference genome used by each laboratory (GRCh37/hg19 or GRCh38/hg38) to identify single-nucleotide variants and small insertions and deletions. Population frequencies were assessed using databases including gnomAD, ExAC, and 1000 Genomes et al. Where specified in the laboratory workflow, variants with population allele frequencies >1% were excluded during initial filtering. Coding variants and variants with potential splice effects were prioritized for further evaluation.

Candidate variants were assessed according to their predicted molecular consequences, population frequencies, phenotype–gene associations, and compatibility with the expected inheritance pattern. OMIM was used to assess phenotypic relevance, while the published literature was reviewed for previous reports and supporting evidence. Candidate *SLC52A2* variant pairs were evaluated for compatibility with autosomal recessive inheritance. The variants were confirmed by Sanger sequencing, and available parental samples were tested to establish segregation and determine whether paired heterozygous variants were in trans.

*SLC52A2* variants were described relative to the reference transcript NM_024531.5. Annotation included nucleotide and protein changes, variant type, zygosity, population frequency, previous reports, conservation, and protein location. For the present study, missense variant effects were assessed using REVEL [29], SIFT, PolyPhen-2, and AlphaMissense [30]. CADD PHRED scores [31] were obtained for the investigated variants, and phyloP scores were used to assess nucleotide conservation. Available prediction scores and classifications are provided in Supplementary Table S2. Variant pathogenicity was classified according to the ACMG/AMP guidelines [32], integrating population, segregation, clinical, and computational evidence.

Structural modeling was performed as an adjunctive in silico analysis. The amino acid positions affected by missense variants in *SLC52A2*/RFVT2 were visualized in the predicted protein structure using PyMOL (version 3.1.3.1; Schrödinger, LLC, New York, NY, USA). Local residue interactions before and after amino acid substitution were predicted using DynaMut2. Interaction types were interpreted according to the DynaMut2 color legend, including clash, van der Waals, hydrogen-bond, ionic, aromatic, hydrophobic, carbonyl and polar contacts. For variants expected to alter RFVT2 length, including nonsense, frameshift and in-frame deletion variants, mutant protein models were generated with SWISS-MODEL and visualized in PyMOL to assess predicted truncation, segment loss and conformational changes. These structural analyses were used as supportive evidence to illustrate possible effects on RFVT2 structure and were not used as independent criteria for ACMG/AMP classification.

### 2.4. Treatment and Follow-Up

All patients received individualized riboflavin supplementation after genetic confirmation or strong clinical suspicion of RTD2. The dose was adjusted according to age, body weight, clinical severity and treatment response. We recorded the starting dose, dose escalation, maintenance dose, body weight-adjusted dose (mg/kg/day), treatment duration and adverse reactions. Patient 1 also received a mitochondrial cocktail in addition to riboflavin, including ethyl polyenoate, phosphatidylserine, levocarnitine and coenzyme Q10. Rehabilitation training, acupuncture, massage, and other symptomatic supportive treatments were provided according to each patient’s clinical condition.

Follow-up was conducted through outpatient visits and telephone interviews. The main assessments included changes in anemia, ataxia, muscle strength, motor function, visual response, abnormal eye movements, respiratory symptoms and hearing-related manifestations. Ataxia severity was assessed using the Scale for the Assessment and Rating of Ataxia (SARA). Overall treatment response was assessed semi-quantitatively using a caregiver-reported visual analog scale (VAS). Caregivers rated the child’s overall symptom severity on a scale from 0 to 10, with 0 indicating no symptoms and 10 representing the most severe pretreatment state. Available pretreatment and post-treatment SARA and VAS scores were recorded alongside clinical changes to assess improvement following riboflavin supplementation and combined treatment. For patients with continuous follow-up data, we also recorded hemoglobin recovery, motor milestone changes, gait stability, upper limb and distal muscle strength, visual and auditory improvement, and symptom recurrence after dose reduction or treatment withdrawal.

### 2.5. Literature Search and Geographic Variant Analysis

To summarize the reported *SLC52A2* variant spectrum and describe its distribution among published cases, we searched PubMed, Web of Science, and Embase from database inception through Feb 2026 using database-specific combinations of *SLC52A2*, RFVT2, “riboflavin transporter 2”, “riboflavin transporter deficiency type 2”, RTD2, and “Brown-Vialetto-Van Laere”. We also screened the reference lists of relevant reports.

Eligible publications included case reports, case series, and original studies describing patients with homozygous or compound heterozygous *SLC52A2* variants identified as the molecular basis of RTD2. No restrictions were applied with respect to age, sex, or geographic origin. Eligible phenotypes included auditory, motor, sensory, ocular, bulbar, respiratory, and hematological manifestations. Hearing impairment was not required, and atypical presentations were eligible when supported by clinical and genetic evidence. Studies were required to provide an identifiable patient-level genotype and a minimum clinical description; complete audiological, treatment, and follow-up data were not required. We excluded cases without documented biallelic *SLC52A2* variants, unaffected carriers, and cases attributed solely to other genes. Reviews without original cases, non-human studies, and reports lacking patient-level genotype–phenotype linkage were also excluded. When the same patient was reported more than once, we retained the most complete or most recent record.

Extracted variables included geographic region, sex, age at onset, hearing phenotype, organ involvement, variant location and type, riboflavin treatment, and cochlear implantation outcomes. Published variants were combined with those identified in our four patients to characterize variant types, recurrent variants, and geographic distribution. Given the rarity of RTD2 and heterogeneity in clinical reporting, all analyses were descriptive, without formal between-group hypothesis testing.

## 3. Results

### 3.1. Clinical Characteristics

**Figure 2:**
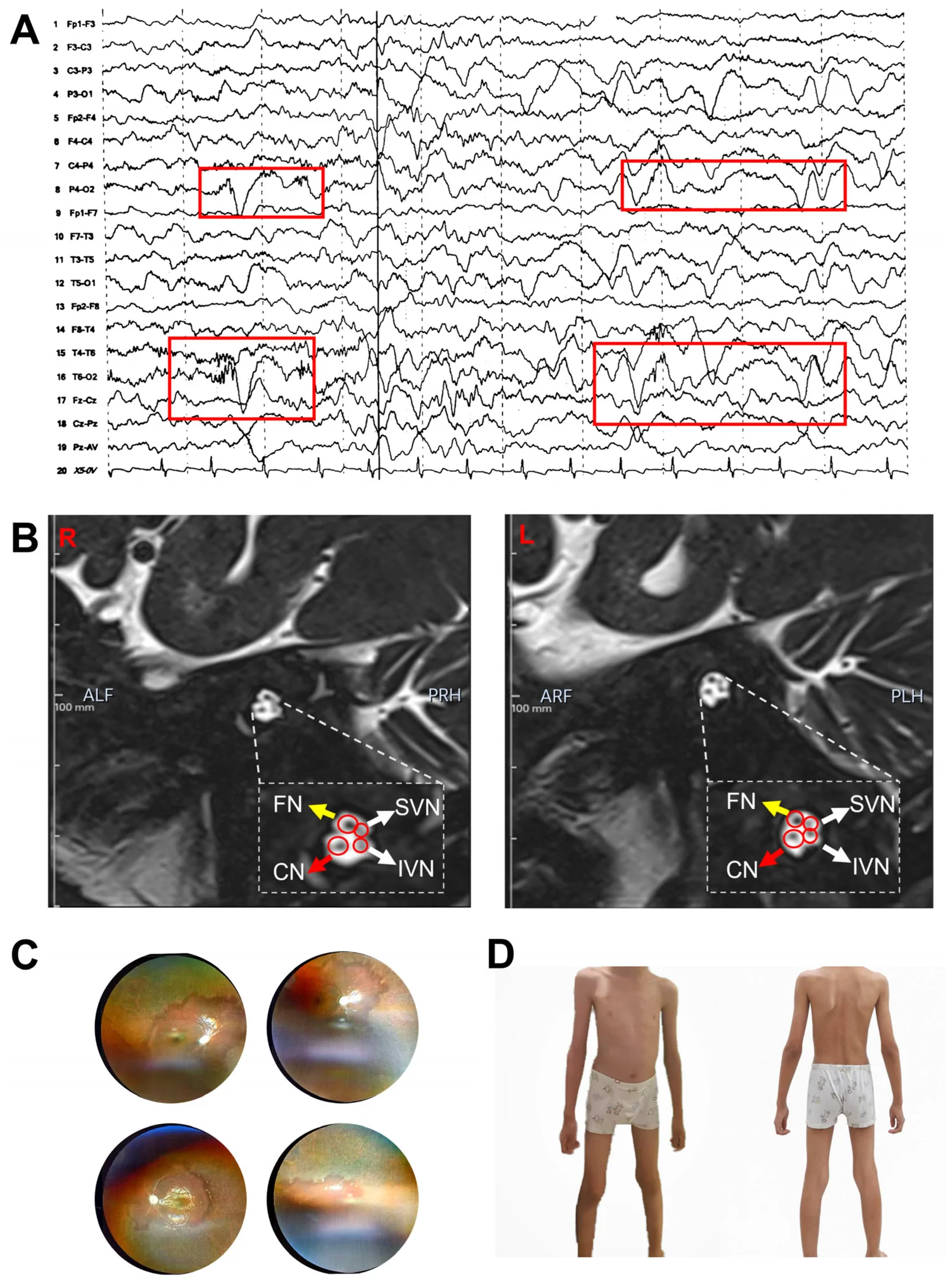
Representative neurological, radiological, motor and ocular findings in patients with *SLC52A2*-related RTD2. (**A**) Sleep electroencephalography of patient 1 showed recurrent irregular sharp-and-slow wave complexes in the right occipital and posterior temporal regions. Red boxes indicate representative abnormal discharges. (**B**) High-resolution MRI of the bilateral internal auditory canals in patient 1 showed no apparent abnormalities of the cochlear nerve or other visible nerve branches. FN, facial nerve; CN, cochlear nerve; SVN, superior vestibular nerve; IVN, inferior vestibular nerve. (**C**) Representative fundus photographs showed optic atrophy in patient 3. (**D**) Clinical photographs of patient 3 showed abnormal posture and motor involvement, consistent with delayed motor development and limb weakness.

All four patients in this study were male and developed symptoms during infancy or early childhood. The main initial or early manifestations were hearing loss, delayed motor development, severe anemia, lower limb weakness, and ataxia (Table 1). The timing of hearing loss differed from that of other systemic symptoms. Patient 1 had the most severe phenotype. At six months of age, he developed bilateral hearing loss, choking during feeding and delayed motor development. He later developed recurrent infectious pneumonia, optic atrophy, nystagmus, anemia, and global developmental delay, as assessed by the Gesell Developmental Schedules. Electroencephalography showed sharp-and-slow wave complexes in the right occipital and posterior temporal regions during sleep (Figure 2A). Inner ear MRI showed no apparent vestibulocochlear nerve developmental abnormalities (Figure 2B). Patient 2 presented with severe bilateral hearing loss at 4 years of age, but subsequent telephone follow-up was unavailable. Patient 3 first developed severe anemia between 10 and 15 months of age, with a lowest hemoglobin level of 54 g/L, followed by lower limb weakness and inability to walk independently. He also had optic atrophy (Figure 2C) and abnormal right visual evoked potentials. Pulmonary function testing suggested moderate obstructive ventilatory dysfunction. Patient 4 developed severe anemia at 2 months of age, with a lowest hemoglobin level of 29 g/L. Delayed motor development gradually became evident after 6 months of age (Figure 2D). After 1 year of age, ataxia worsened and was accompanied by optic atrophy, nystagmus, amblyopia and bilateral mild to moderate hearing loss. Cranial MRI showed bilateral symmetric punctate T2 hyperintensities in the dorsal midbrain and pons, with bilateral ventricular enlargement. Cervical spinal MRI showed abnormal signals. Electromyography suggested multiple peripheral neuropathy involving motor and sensory fibers. Overall, the four patients showed core features of RTD2, including early onset, hearing loss or auditory neuropathy, motor involvement, ocular involvement and hematologic abnormalities. Patients 1 and 3 also had respiratory involvement, suggesting that respiratory status should be monitored during follow-up in patients with *SLC52A2*-related disease.

### 3.2. Audiological Phenotypes

**Figure 3:**
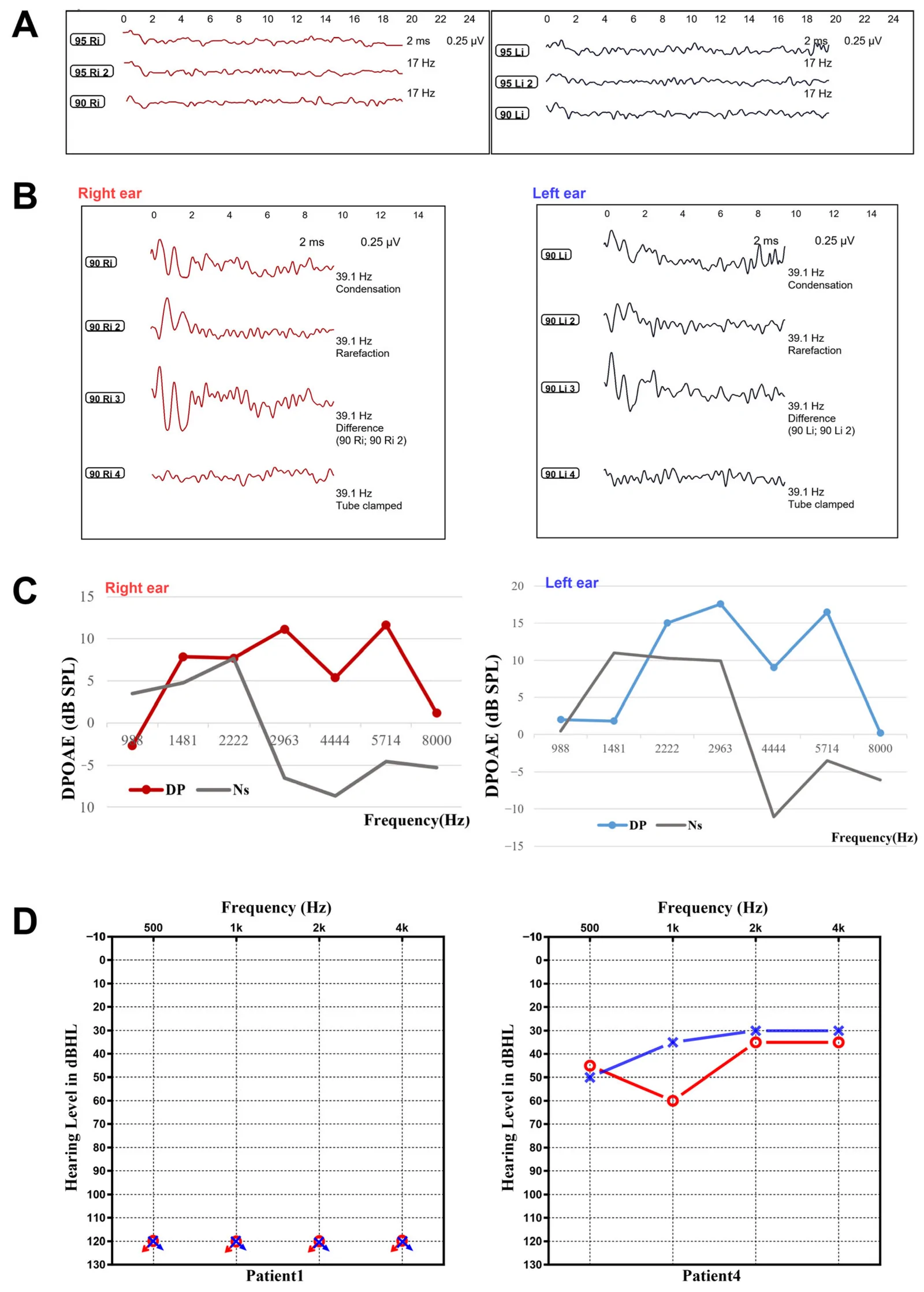
Representative audiological phenotypes of patients with *SLC52A2*-related RTD2. Results are from Patient 1 unless otherwise indicated. (**A**) ABR waveforms were absent at 95 dB nHL in both ears. (**B**) CM responses were recorded bilaterally. (**C**) DPOAEs were absent at 988, 1481 and 2222 Hz in both ears but were present at the other tested frequencies. (**D**) Behavioral audiometry showed variable degrees of hearing loss among patients. Patient 1 had bilateral profound hearing loss, whereas Patient 4 had bilateral mild to moderate hearing loss. Red, right ear; blue, left ear. ABR, auditory brainstem response; CM, cochlear microphonic; DPOAEs, distortion product otoacoustic emission.

Three patients met the electrophysiological diagnostic criteria for typical auditory neuropathy (Figure 3). Patients 1, 2 and 4 had absent ABR waveforms under high-intensity acoustic stimulation (Figure 3A), whereas bilateral CM responses were preserved (Figure 3B). Although Patient 3’s hearing did not significantly interfere with daily communication or learning, his ABR showed poorly differentiated waveforms. These findings were consistent with impaired auditory nerve synchrony and relatively preserved cochlear microphonic potentials. In Patients 1 and 4, DPOAEs were still detectable at some frequencies in both ears (Figure 3C), further supporting partial preservation of outer hair cell function. In Patient 2, DPOAEs were absent bilaterally, but preserved bilateral CM responses remained consistent with auditory neuropathy spectrum disorder. Acoustic immittance showed type A tympanograms in both patients, with no evidence of a middle ear conductive component. Behavioral audiometry showed bilateral profound hearing loss in Patients 1 and 2 and bilateral mild to moderate hearing loss in Patient 4 (Figure 3D). These findings suggest that the auditory phenotype of *SLC52A2*-related RTD2 is heterogeneous and may range from mild or moderate hearing loss to profound hearing loss.

### 3.3. SLC52A2 Genotypes and Variant Interpretation

All four patients carried biallelic variants in *SLC52A2*. Eight variants were identified in total (Table 2), comprising missense, nonsense, frameshift, and in-frame deletion variants. Four of these variants, c.1247C>T, c.75_76insCCTGG, c.887C>T, and c.568C>T, have not been reported previously.

The frameshift variant p.A26Pfs*42 and the nonsense variant p.W198* are predicted to introduce premature termination codons, resulting in protein truncation and/or nonsense-mediated mRNA decay. Because loss of *SLC52A2*/RFVT2 function is an established mechanism of RTD2, these variants support a likely pathogenic interpretation (Table 2). The missense variants and in-frame deletions require integrated assessment of population frequency, computational prediction, conservation, family co-segregation and phenotype consistency. Although several variants remain classified as variants of uncertain significance, the biallelic genotype, highly consistent multisystem phenotype, and clinical response to riboflavin collectively provide strong clinical support for RTD2.

**Figure 4:**
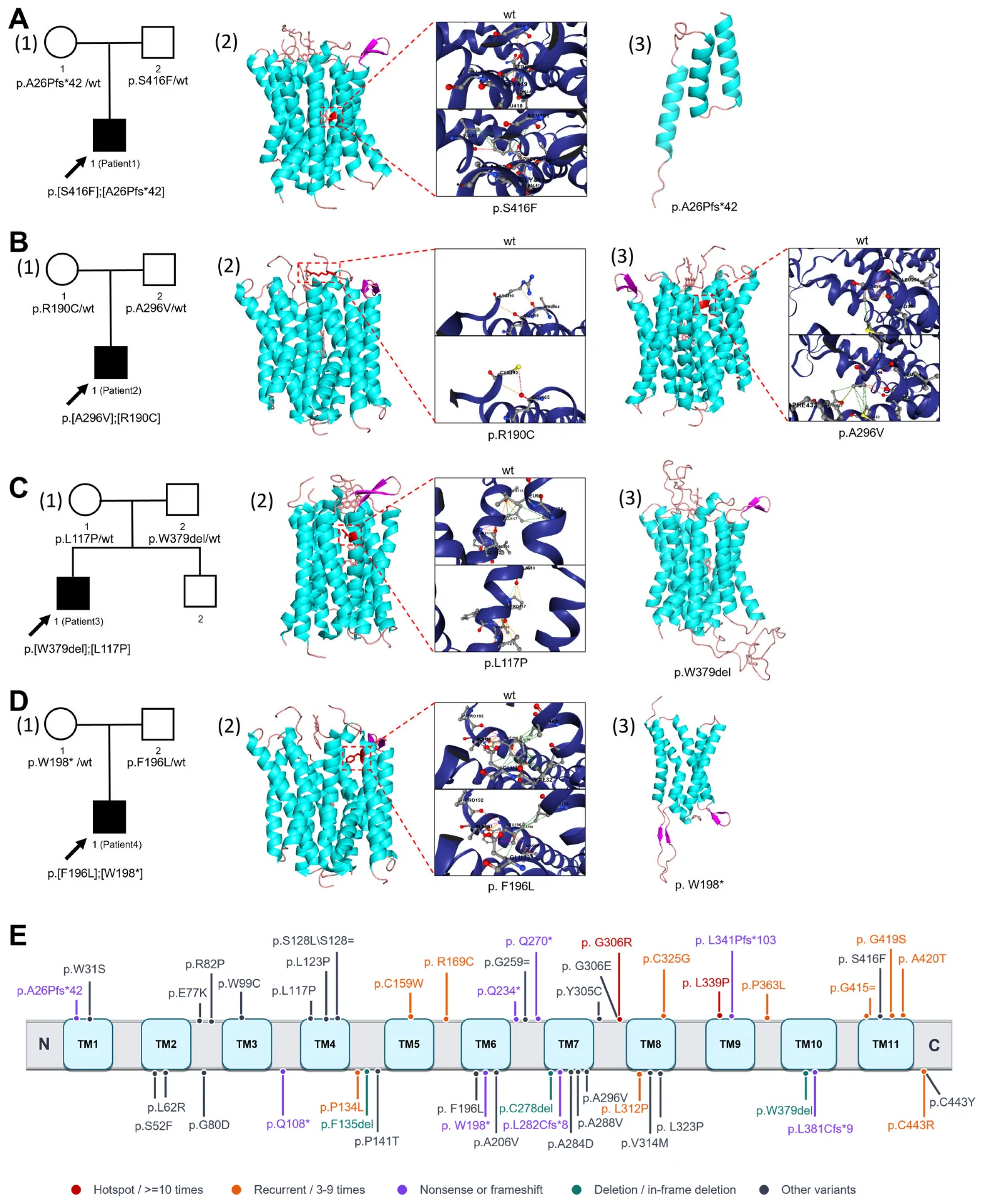
Pedigree analysis and predicted structural effects of *SLC52A2* variants in the four patients. (**A**–**D**) Pedigrees and structural prediction results for Patients 1–4, respectively. In each panel, (**1**) shows family segregation of the compound heterozygous *SLC52A2* variants; (**2**), (**3**) show local residue-interaction changes for missense variants and predicted conformational effects for variants causing truncation, deletion or protein-length alteration. (**A**) Patient 1 carried p.S416F and p.A26Pfs*42. (**B**) Patient 2 carried p.A296V and p.R190C. (**C**) Patient 3 carried p.W379del and p.L117P. (**D**) Patient 4 carried p.F196L and p.W198*. (**E**) Schematic distribution of *SLC52A2* variants across the predicted 11-transmembrane-domain topology, with variants detected in this cohort and previously reported recurrent variants indicated.

Pedigree analysis showed that all four probands carried compound heterozygous *SLC52A2* variants inherited from clinically unaffected carrier parents, consistent with autosomal recessive inheritance (Figure 4A–D).

Computational predictions varied among the missense variants (Supplementary Table S2). Predictions for p.S416F consistently supported a deleterious effect, with REVEL, SIFT, PolyPhen-2, and CADD PHRED scores of 0.841, 0, 1.000, and 27.9, respectively, and an AlphaMissense classification of likely pathogenic (0.7465). Predictions for the other variants were less consistent. AlphaMissense classified p.A296V, p.R190C, and p.L117P as likely benign, whereas p.F196L was classified as likely pathogenic (0.9533) despite a SIFT score of 0.65. For the variants that altered protein length, CADD PHRED scores were 29.7 for p.A26Pfs*42, 40 for p.W198*, and 20.3 for p.W379del. Structural modelling illustrated the predicted effects of these variants on RFVT2 (Figure 4A–D). DynaMut2 models showed changes in local non-covalent interactions around all five missense substitutions, although these changes did not establish impaired protein folding or stability. SWISS-MODEL and PyMOL models predicted that p.A26Pfs*42 would leave only a short N-terminal fragment and that p.W198* would remove the downstream transmembrane region. The p.W379del model showed a local structural change while retaining the overall transporter scaffold. These structural predictions require further functional validation.

The variants were distributed across transmembrane and loop regions of RFVT2, with no apparent clustering at known recurrent hotspots (Figure 4E). Based on the annotated topology, p.L117P, p.A296V, and p.S416F were located in TM4, TM7, and TM11, respectively; p.F196L lay at the start of TM6, and p.R190C in the loop between TM5 and TM6. The in-frame deletion p.W379del affected TM10, whereas p.A26Pfs*42* and p.W198 were predicted to remove downstream transmembrane segments. S416 is part of a previously reported hydrogen-bond network connecting the N- and C-terminal domains of RFVT2 [6]. In the DynaMut2 models, p.S416F altered local polar contacts and introduced additional hydrophobic contacts around F416. The p.R190C model showed a predicted hydrogen-bond contact involving the sulfur atom of C190, while p.A296V and p.F196L showed changes in local hydrogen-bond contacts. The p.L117P and p.F196L models also displayed fewer hydrophobic contacts than their wild-type counterparts (Figure 4A–D). These models suggest changes in local interactions, but their effects on transporter activity and conformational stability remain to be established.

### 3.4. Response to Riboflavin Treatment

Patients 1, 3, and 4 received riboflavin supplementation and showed varying degrees of clinical improvement after treatment. After treatment, the median family-reported VAS score for overall symptom severity decreased from 10 to 5, and the median SARA score decreased from 28 to 14 among the three assessed patients (Table 1). Patient 1 started riboflavin treatment at 1 year of age, with an initial dose of 60 mg/day. Hemoglobin levels normalized after about 6 months. The dose was then increased and maintained at 400 mg/day, approximately 40 mg/kg/day. Visual responsiveness, motor ability, and involuntary upward eye deviation improved. At 1 year and 11 months, the dose was reduced to 200 mg/day, approximately 20 mg/kg/day, and mitochondrial cocktail therapy was added. During dose reduction to 250 mg/day, involuntary upward eye deviation recurred and the patient developed pneumonia. After riboflavin was increased again to 400 mg/day, the disease course became more stable. The child was able to stand and walk with support, although improvement in hearing and vision remained limited. Patient 3 had the mildest phenotype and appeared particularly responsive to riboflavin. He started riboflavin at 3 years of age, with an initial dose of 20 mg/day, approximately 1.7 mg/kg/day, and was able to stand independently after 3 months. He has been maintained on 80 mg/day, approximately 4.5 mg/kg/day, for 1 year and currently attends school. His ataxia improved, and he can walk independently with a relatively stable gait. Muscle strength also improved, although distal weakness persisted. Muscle strength in both upper limbs was grade 3 overall, with right distal muscle strength of grade 1 and left distal grip strength of grade 2. He also had scoliosis and bilateral optic atrophy, which was more severe on the right side; he therefore relied mainly on the left eye for vision. In addition to riboflavin, he received acupuncture and massage rehabilitation from 2.5 years of age. After rehabilitation, upper limb strength and nystagmus showed partial improvement. Patient 4 started riboflavin after diagnosis at 2 years of age. The initial dose was 10 mg/day and was increased to 35 mg/day after 7 days, approximately 3 mg/kg/day. Hemoglobin normalized within 7 days. The dose was increased to 160 mg/day at 2.5 years of age and to 600 mg/day at 4 years of age. He has remained on this dose to date, approximately 37.5 mg/kg/day. After treatment, ataxia, gait stability, and upper limb muscle strength improved. However, distal muscle weakness, optic atrophy, and mild to moderate hearing loss persisted. Overall, riboflavin treatment showed clear benefits for hematologic abnormalities, motor function and ataxia, but its effect on hearing and vision was limited in patients with established auditory nerve or optic nerve damage.

### 3.5. Exploratory Geographic Distribution of SLC52A2 Variants

The literature review identified 34 eligible studies reporting 87 patients with *SLC52A2*-related RTD2, encompassing 52 distinct *SLC52A2* variants (Table S1). These previously reported cases were used to characterize the global variant spectrum and to contextualize the four Chinese patients in the present cohort (Figure 1).

**Figure 5:**
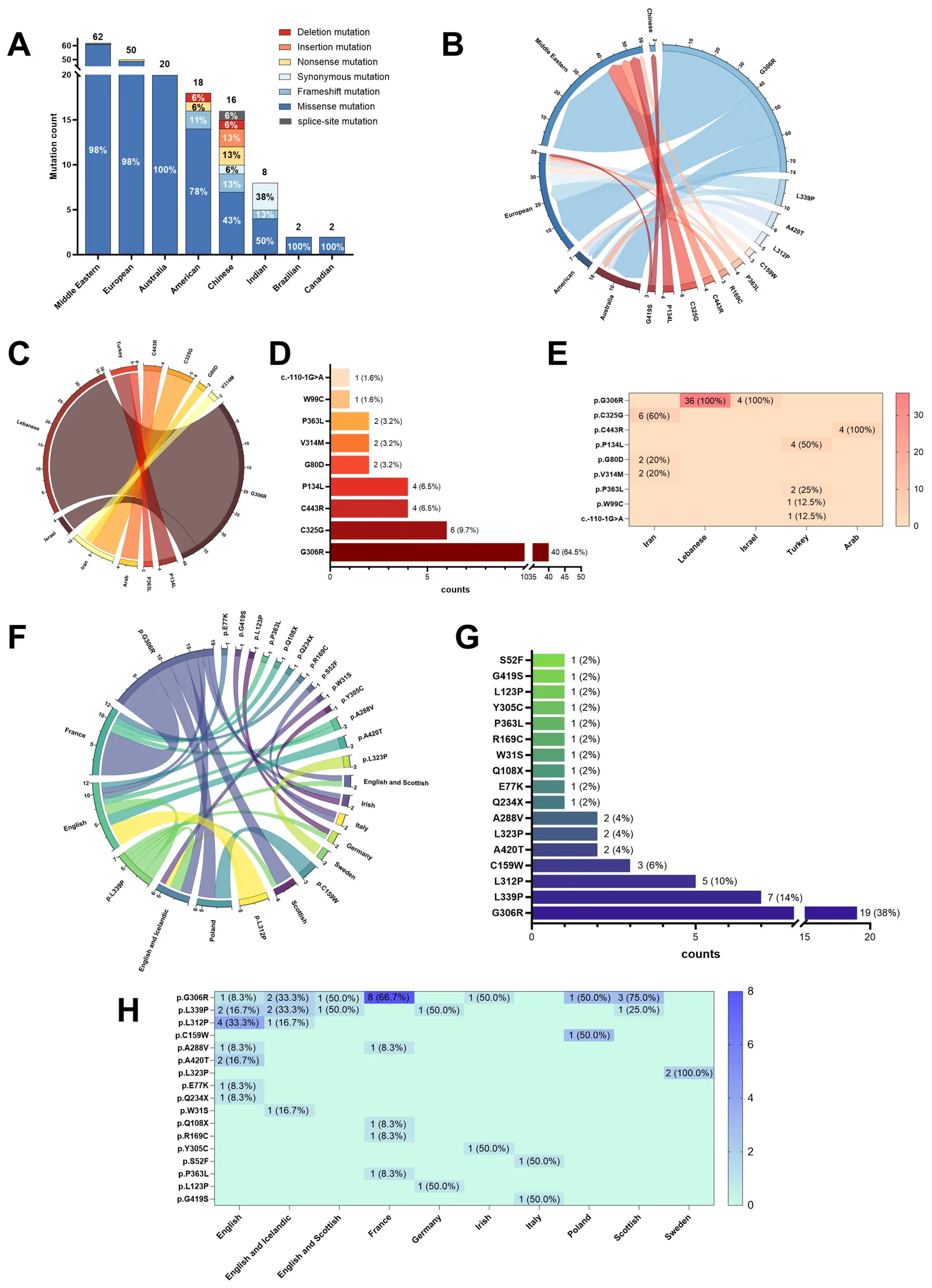
Reported geographic distribution of *SLC52A2* variant alleles in the assembled case dataset. (**A**) Proportion of different variant types by region. (**B**) Chord diagram showing the geographic distribution of recurrent variants represented more than three times in the dataset. (**C**) Chord diagram showing the reported allele counts of different *SLC52A2* variants by reported geographic/ancestry category in the Middle East. (**D**) Variant sites and occurrence counts in the Middle East. (**E**) Heat map of variant sites and occurrence counts in Middle Eastern countries. (**F**) Chord diagram showing the reported allele counts of different *SLC52A2* variants by reported geographic/ancestry category in Europe. (**G**) Variant sites and occurrence counts in Europe. (**H**) Heat map of variant sites and occurrence counts in European countries.

In the integrated allele-level analysis, missense variants represented the predominant variant type, accounting for 89.3% (159/178) of all variant alleles analyzed. Missense variants were particularly predominant across the major geographic populations represented in the dataset, while other variant types, including nonsense, frameshift, splice-site, synonymous, and in-frame deletion variants, were less frequently reported (Figure 5A). The p.G306R variant was the most frequent recurrent variant, accounting for 41.6% (74/178) of all analyzed variant alleles, and was mainly reported in patients from the Middle East, Europe, and Australia (Figure 5B). A founder effect of p.G306R has previously been described in Lebanese [14] and Israeli populations (Figure 5C). In addition, p.G306R in either homozygous or compound heterozygous states has been reported in multiple families from France, Australia, Israel, Scotland, England, and the United States.

Further analysis by region showed that the variant spectrum in Middle Eastern populations was relatively concentrated (Figure 5C,F). In the included Middle Eastern reports, p.G306R accounted for 64.5% of reported alleles (40/62; Figure 5C,D). Lebanese and Israeli cases included in this review carried p.G306R, whereas three variant sites were reported in the Iranian cases (Figure 5E). Among the European reports, 17 variant sites were identified, 11 of which occurred only once (Figure 5F). The p.G306R variant accounted for 38.0% of reported alleles in this group (19/50; Figure 5G). The European reports also included p.L339P in individuals described as having English or mixed English and Icelandic ancestry (Figure 5H). These counts reflect the composition of the published cases and should not be interpreted as population-specific allele frequencies, ethnic specificity, or differences in genetic heterogeneity. Family clustering, reporting practices, and access to genetic testing may have influenced the observed distributions.

Unlike the hotspot variants repeatedly reported in previous studies, no Chinese patient in the current dataset, including the four patients in this study and previously reported Chinese cases, carried p.G306R, p.L339P, or p.A420T. The eight variants identified in our four patients were distributed across different regions of *SLC52A2* and included missense, nonsense, frameshift, and in-frame deletion variants. In the available Chinese dataset, the variant distribution appeared more dispersed than the hotspot-dominated patterns observed in Middle Eastern and European reports.

## 4. Discussion

This study used non-isolated auditory neuropathy as the clinical entry point for identifying *SLC52A2*-related RTD2. The four Chinese patients with biallelic *SLC52A2* variants all presented with hearing abnormalities. In two patients, hearing loss was the first or co-initial manifestation. In the other two, severe anemia, motor delay or ataxia preceded or outweighed hearing complaints in the early disease course. The order of symptom onset varied across the four patients. Audiologists and pediatric specialists should therefore consider RTD2 when auditory neuropathy co-occurs with anemia, motor delay, optic atrophy, or respiratory symptoms. When auditory neuropathy is early onset, progressive or accompanied by extra-auditory signs, *SLC52A2* should be included in the differential diagnosis and genetic testing should be performed without delay.

All four patients carried biallelic *SLC52A2* variants, with eight variants identified in total. Four variants detected in patients 1 and 2 had not been reported previously, including missense variants and one frameshift truncating variant. The frameshift variant p.A26Pfs*42 is predicted to introduce a premature protein termination or trigger nonsense-mediated mRNA decay [33], consistent with loss of RFVT2 function. Patient 2 carried two novel missense variants, p.A296V and p.R190C. Patient 2’s auditory phenotype and segregation findings support consideration of RTD2, but systemic characterization and treatment response were unavailable. These findings extend the known *SLC52A2* variant spectrum in Chinese patients and highlight the need to integrate genotype, phenotype and familial segregation while retaining uncertainty in variant interpretation.

Auditory neuropathy was a useful clinical clue linking these patients to RTD2, but it should be interpreted as part of a multisystem presentation and is not diagnosis-defining on its own. Typical auditory neuropathy is characterized by absent or markedly abnormal ABR, with preservation of CM and/or DPOAEs in the early stage, indicating impaired neural conduction with relatively preserved outer hair cell function [34]. Patients 1 and 2 were referred because of profound hearing loss. Their audiological tests showed absent ABR and recordable CM, and Patient 1 also had preserved DPOAEs. These findings support early genetic testing in children with non-isolated auditory neuropathy, especially when motor delay, optic atrophy, anemia, ataxia or respiratory manifestations are present.

Respiratory involvement was also observed in this cohort. Patient 1 had recurrent infectious pneumonia, and Patient 3 had moderate obstructive ventilatory dysfunction. These findings are consistent with previous reports and indicate that respiratory assessment and supportive care should be included in long-term follow-up. In a review of 109 patients with RTDs, respiratory failure was the main cause of death in untreated patients [19]. Another retrospective study reported respiratory involvement in up to 57% of patients with RTD2 and 76% of patients with RTD3, assisted ventilation was required, respectively, in 65% and 41% of these patients [10]. A case report further showed that, even with high-dose riboflavin treatment, respiratory insufficiency and related pneumonia may remain the immediate cause of death [35]. During follow-up, swallowing function, vocal cord mobility, diaphragmatic function, nocturnal ventilation, and pulmonary function should be assessed regularly. Non-invasive ventilation, airway clearance, nutritional support, and rehabilitation should be initiated when clinically indicated [36].

The phenotypic differences in this cohort may partly reflect a genotype–phenotype correlation in *SLC52A2*-related RTD2. Patient 1 had the most severe phenotype, with bilateral hearing loss, feeding difficulty, and delayed motor development by six months of age, followed by recurrent pneumonia, optic atrophy, nystagmus, anemia, and global developmental delay. The truncating variant p.A26Pfs*42 may cause substantial loss of RFVT2 function and contribute to his early onset and multisystem involvement. In contrast, patients 3 and 4 showed clearer motor improvement after riboflavin treatment, suggesting that variant type, residual transporter function, and treatment timing may influence clinical outcome. Previous studies have also suggested that *SLC52A2* variant type may influence clinical presentation. Patients with splice-site variants may have earlier and more severe onset. For example, a previously reported patient carrying compound heterozygous *SLC52A2* variants, including one splice-site variant and one missense variant, developed widespread muscle weakness and respiratory dysfunction shortly after birth and required mechanical ventilation [37]. Among patients with missense variants, homozygous variants have been associated more frequently with ataxia as the initial manifestation, whereas compound heterozygous variants may be associated with respiratory insufficiency during the disease course [14]. Overall, bilateral sensorineural deafness and ataxia are common early manifestations of RTD2 [14,38,39]. Other features may include muscle weakness, optic atrophy, nystagmus, and respiratory involvement. In the early stage, upper limb weakness may be more prominent than lower limb weakness. With disease progression, lower limb and axial weakness can worsen and eventually lead to loss of independent ambulation [38]. Because the number of reported cases remains limited, the relationship between *SLC52A2* variant type, residual protein function, and clinical phenotype requires further functional validation and larger cohort studies.

Riboflavin supplementation is the main treatment for RTD2, and because humans cannot synthesize riboflavin, treatment should be started as soon as RTD2 is suspected and generally continued lifelong [40]. Daily riboflavin doses have ranged from 2.0 to 80.0 mg/kg [4]. In our cohort, overall symptom burden decreased after treatment, with VAS scores decreasing from 10 to 5. Improvement was more evident in anemia, ataxia, muscle strength and motor function than in hearing or vision. This differential treatment response is relevant to the timing of clinical suspicion: symptoms that appear before or alongside hearing loss may remain more modifiable than established auditory or optic nerve injury. Previous studies have reported clinical improvement in approximately half of treated patients [36], with gross motor function, bulbar palsy, and ataxia appearing to be among the most treatment-responsive features [41]. Given the saturable and transporter-mediated nature of riboflavin uptake at the blood–brain barrier, it remains possible that systemic riboflavin exposure influences central nervous system bioavailability in RTD2. In patients retaining partial RFVT2 activity, higher or more sustained plasma riboflavin concentrations may partially compensate for impaired transporter function through substrate-driven transport [42,43]. However, current clinical data have not established a body-weight-adjusted minimum effective dose or plasma concentration required for adequate CNS delivery [41].

For patients with RTD2-associated severe or profound hearing loss who derive insufficient benefit from hearing aids, cochlear implantation may be considered. Given the prominent peripheral and cranial neuropathy associated with RTD2 [13], *SLC52A2*-related auditory neuropathy is thought to predominantly involve postsynaptic neural structures rather than a purely presynaptic cochlear lesion. Preserved CM and/or DPOAEs with absent or markedly abnormal ABRs suggest impaired neural transmission. Among the published cases summarized in Table 3, hearing thresholds improved after implantation in some patients, whereas speech perception and language comprehension remained limited [4,18,44]. Patient 1 underwent right cochlear implantation; however, no measurable intraoperative responses were recorded across the 24 tested electrodes. This may reflect dysfunction of the auditory nerve, retrocochlear pathway or central auditory system [45]. According to the clinical practice guideline for auditory neuropathy, outcomes in postsynaptic auditory neuropathy are more variable and tend to be less favorable [27]. Some patients may achieve only sound awareness, with limited functional speech perception. Candidate evaluation should therefore extend beyond hearing thresholds and incorporate audiological findings, cochlear nerve imaging, disease progression, duration of auditory deprivation, communication needs, and rehabilitation potential [46]. Families should be counselled that implantation may improve sound awareness but may not restore satisfactory speech recognition when auditory nerve or central pathway damage is present [47,48]. Further studies are required to identify predictors of cochlear implant outcomes in postsynaptic auditory neuropathy.

Riboflavin treatment cannot fully reverse established nervous system injury, so additional therapeutic strategies are still needed. Some patients deteriorate or die suddenly despite continuous riboflavin supplementation [4,8,35]. One possible reason is that RTD2 may involve mechanisms beyond riboflavin and flavin cofactor deficiency [3,49]. RFVT2 is mainly expressed in the brain and spinal cord [50,51]. Its misfolding can induce endoplasmic reticulum stress and mitochondrial dysfunction early in the disease [52], whereas exogenous riboflavin is unlikely to repair fixed structural injury in the nervous system [10,17]. Consistent with its essential biological role, complete loss of *Slc52a2* is embryonic lethal in mice [53]. Gene therapy is therefore under investigation. In vitro studies showed that AAV9-mediated delivery of codon-optimized human *SLC52A2* cDNA improved neurite outgrowth and neural network stability in patient-derived motor neurons [54]. This strategy remains experimental, and further studies are required to evaluate safety, tissue targeting, long-term expression, and clinical feasibility.

*SLC52A2* variant spectrum appears to differ by region. The p.G306R variant was the most frequently reported hotspot and had been repeatedly identified in Lebanese and Israeli patients, consistent with a founder effect in these populations [8,55,56,57]. None of the Chinese patients in this study carried classic hotspot variants, such as p.G306R, p.L339P, or p.A420T. Instead, their variants were distributed across different regions of *SLC52A2*. These findings suggest a more dispersed variant spectrum in Chinese patients. Therefore, suspected RTD2 in Chinese patients should not be assessed only by hotspot screening. Sequencing should cover all coding regions and splice regions of *SLC52A2*.

## 5. Limitations

This study has several limitations. First, RTD2 is extremely rare, which limited the number of eligible patients. This retrospective case series included only four patients (*n* = 4) from an auditory neuropathy cohort. The small sample size and hearing-based ascertainment limit generalizability and do not allow robust analyses of genotype–phenotype associations or geographic distribution. Second, audiological data were heterogeneous. This is expected in a retrospective real-world study, in which testing is guided by clinical needs rather than a standardized protocol. In Patient 3, systemic manifestations were more prominent than hearing impairment; therefore, only partial audiological evaluation was performed. Patient 2 was lost to long-term follow-up, limiting the available information on systemic manifestations. Third, several variants identified in the four patients remain classified as VUS-favor LP. In silico predictions and structural modeling provide supportive evidence but cannot replace functional validation. Cellular studies are needed to determine whether these variants impair riboflavin transport. Finally, the literature-based analysis may be affected by publication and ascertainment bias, overlapping or related cases, incomplete reporting, and uncertainty in geographic or ancestry assignment. Geographic patterns and genotype–phenotype associations should therefore be considered exploratory.

## 6. Conclusions

In summary, *SLC52A2*-related RTD2 should be considered in children with early-onset or progressive non-isolated auditory neuropathy, particularly when hearing abnormalities are accompanied by delayed motor development, ataxia, optic atrophy, anemia or respiratory involvement. In this study, four patients with *SLC52A2*-related RTD2 were identified from a Chinese auditory neuropathy cohort. Hearing loss was the first or co-initial manifestation in two patients. Riboflavin supplementation was associated with improvement in hematologic abnormalities, ataxia, muscle strength, and motor function in some patients, whereas established auditory and visual impairment showed limited reversibility. These findings support early molecular diagnosis and timely riboflavin treatment to improve clinical outcomes. The *SLC52A2* variant spectrum also appeared to differ across geographic populations. The variants identified in Chinese patients differed from the hotspot-dominant pattern reported in Middle Eastern and European cohorts. Larger multicenter studies with longer follow-up are needed to clarify geographic and genotype–phenotype patterns, as well as the effects of treatment timing and riboflavin dose. In parallel, gene therapy may represent a promising future strategy for *SLC52A2*-related RTD2 and warrants further preclinical investigation.

## Figures and Tables

**Table 1:** Clinical and audiological characteristics of patients with *SLC52A2*-related disease.

Patient ID	Patient 1	Patient 2	Patient 3	Patient 4
Sex	Male	Male	Male	Male
Current age	2 years 6 months	13 years 3 months	8 years 11 months	4 years 11 months
BMI (kg/m^2^)	15.55	NA	11.34	13.72
Age at onset	6 months	4 years	10 to 15 months	2 months
Initial or early manifestations	Hearing loss, choking, and motor developmental delay	Hearing loss	Severe anemia, followed by lower-limb weakness and inability to walk independently	Severe anemia, followed by motor developmental delay and ataxia
Auditory phenotype	Bilateral severe hearing loss; bilateral ABR threshold >95 dB nHL; DPOAEs elicited at some frequencies; bilateral CM present	Bilateral severe hearing loss; bilateral ABR threshold >100 dB nHL; bilateral CM present	Bilateral elevated hearing thresholds and poorly differentiated ABR waveforms	Bilateral mild-to-moderate hearing loss; bilateral ABR thresh-old >95 dB nHL; DPOAEs elicited at some frequencies; bilateral CM present
Ocular manifestations	Optic atrophy, nystagmus, and involuntary upward eye deviation	NA	Optic atrophy	Optic atrophy, nystagmus, and amblyopia
Neurological manifestations	Motor developmental delay, walking difficulty, right facial spasm, and abnormal Gesell developmental assessment	NA	Bilateral lower-limb weakness; previously unable to walk independently; normal cognition	Upper-limb weakness, ataxia, inability to stand independently
Respiratory manifestations	Recurrent infectious pneumonia	NA	Moderate obstructive ventilatory dysfunction	No definite respiratory disorder recorded
Hematological manifestations	Anemia	NA	Severe anemia; previously treated as pure red cell aplasia	Severe anemia; previously treated as pure red cell aplasia
Imaging/electrophysiology	Brain MRI showed widened frontotemporal extracerebral space; later EEG showed abnormal discharges	NA	Brain MRI showed no obvious abnormality; pulmonary function was abnormal; VEP was abnormal on the right side	Brain MRI showed bilateral symmetric punctate T2 hyperintensities in the dorsal midbrain and pons with bilateral ventricular enlargement; cervical MRI showed abnormal spinal cord signals; EMG indicated multiple peripheral neuropathies
*SLC52A2* genotype	c.1247C>T/c.75_76insCCTGG	c.887C>T/c.568C>T	c.1135_1137delTGG/c.350T>C	c.588C>G/c.593G>A
Protein change	p.S416F/p.A26Pfs*42	p.A296V/p.R190C	p.W379del/p.L117P	p.F196L/p.W198*
Riboflavin treatment	Maximum dose approximately 400 mg/day, about 40 mg/kg/day	NA	Currently 80 mg/day, about 4.5 mg/kg/day	Gradually increased to 600 mg/day, about 37.5 mg/kg/day
Treatment response				
SARA score	40 to 34	NA	20 to 5	28 to 14
VAS score	10 to 5	NA	10 to 5	10 to 5
Follow-up outcome	Anemia resolved, partial improvement in visual response and motor ability; symptom recurrence after dose reduction	NA	Marked response; able to stand and walk independently, with improved gait stability	Hemoglobin normalized; ataxia, gait stability, and upper-limb strength improved; mild-to-moderate hearing loss persisted

Note: ABR, auditory brainstem response; BMI, body mass index; CM, cochlear microphonic; DPOAEs, distortion product otoacoustic emissions; EEG, electroencephalography; EMG, electromyography; MRI, magnetic resonance imaging; VEP, visual evoked potential; NA, not available; VAS, visual analog scale; SARA, Scale for the Assessment and Rating of Ataxia.

**Table 2:** *SLC52A2* variants identified in this study.

Patient ID	Nucleotide Change	Protein Change	Variant Type	Pathogenicity Evidence	ACMG Classification	Novel or Reported
Patient 1	c.1247C>T	p.S416F	Missense	PM2_Supporting + PM3 + PP3 + PP4	VUS-favor LP	Novel
Patient 1	c.75_76ins CCTGG	p.A26Pfs*42	Frameshift	PVS1 + PM2_Supporting + PM3 + PP4	LP	Novel
Patient 2	c.887C>T	p.A296V	Missense	PM2_Supporting + PM3_Supporting + PP3 + PP4	VUS-favor LP	Novel
Patient 2	c.568C>T	p.R190C	Missense	PM2_Supporting + PM3_Supporting + PP3 + PP4	VUS-favor LP	Novel
Patient 3	c.1135_1137 delTGG	p.W379del	In-frame deletion	PM2_Supporting + PM4 + PM3_Supporting + PP4	VUS-favor LP	Reported
Patient 3	c.350T>C	p.L117P	Missense	PM2_Supporting + PM3_Supporting + PP3 + PP4	VUS-favor LP	Reported
Patient 4	c.588C>G	p.F196L	Missense	PM1 + PM2_Supporting + PM3 + PP3 + PP4	LP	Reported
Patient 4	c.593G>A	p.W198*	Nonsense	PVS1 + PM2_Supporting + PM3 + PP4	LP	Reported (dbSNP: rs1818749427)

Note: ACMG, American College of Medical Genetics and Genomics; LP, likely pathogenic; VUS, variant of uncertain significance; VUS-favor LP, variant of uncertain significance favoring likely pathogenic.

**Table 3:** Summary of reported RTD2 patients with cochlear implantation.

Authors	This Study	Paprocka et al. [4].	Jreissati et al. [8].	Piecuch et al. [18].	Zhao et al. [14].	Omar et al. [12].	Anderson et al. [44].	Anderson et al. [44].	Menezes et al. [15].	Sinnathuray et al. [45].	Sinnathuray et al. [45].
Year	2026	2025	2025	2023	2022	2021	2019	2019	2016	2011	2011
Sex	M	F	M	F	M	M	F	M	F	M	F
Age at onset of general symptoms	0.5y	3y	2y	2.8y	3.4y	10y	3y	2y	3y	9y	14.5y
Age at onset of hearing loss	0.5y	3y	2y	3.25y	3.5y	0y	2y	3y	9y	9y	14.5y
Mutation points	p.S416F/p.A26Pfs*42	p.G306R/p.C159W	p.G306R/ p.G306R	p.C159W/ p.G306R	p.G419S/ p.G419S	NA	NA	NA	p.G306R/ p.G306R	NA	NA
Symptoms											
Speech delay	+	+	+	+	+	+	+	+	+	+	+
Dysarthria	+	+	+	+	+	+	+	+	+	+	+
Dysphonia	-	-	-	+	-	+	-	-	-	-	-
Motor delay	+	+	+	+	+	+	+	+	+	+	-
Ataxia	+	+	+	+	+	+	+	+	+	+	-
Dysphagia	+	+	-	+	+	++	+	-	+	+	-
Nystagmus	+	-	-	+	+	+	+	+	+	-	+
Optic atrophy	+	+	-	+	+	+	+	-	+	+	+
Visual impairment	+	+	-	+	+	+	+	-	+	+	+
Hypotonia	+	+	+	+	+	+	+	-	+	+	+
Gait abnormalities	+	+	+	+	+	+	+	+	+	+	+
Facial paresis	-	-	-	-	-	-	+	-	-	-	-
Anemia	+	-	-	-	+	-	-	-	-	-	-
Respiratory disorder	+	-	-	-	-	+	-	-	-	+	-
Riboflavin treatment	40 mg/kg	100 mg/kg	25 mg/kg	50 mg/kg	10 mg/kg	NA	400 mg TID	70 mg/kg	1600 mg	NA	NA
Therapy success	Partial	Partial	Partial	Yes	Yes	NA	Partial	Partial	Partial	NA	NA
Cochlear Implantation	Unilateral	Bilateral	Unilateral	Bilateral	Unilateral	Unilateral	Unilateral	Unilateral	Unilateral	Unilateral	Unilateral
Implantation age	2.5y	6y	4.5y	4y	6.8y	17y	8.9y	6.9y	10.5y	41y	45y
Cochlear implant benefit	Very little	Improved thresholds, limited speech differentiation	NA	Hearing improved from profound to moderately severe/severe levels.	NA	Very little, did not wear consistently	Improved thresholds, Single words used	positive to auditory stimulation	significant improvement in speech perception	Hearing thresholds improved but no functional speech recognition	Mild improvement in speech recognition in quiet environments

Note: M, male; F, female; y, years; TID, three times daily; NA, not available. “+” indicates that the symptom was present, “-” indicates that it was absent, and “++” indicates a more severe manifestation as described in the original report. Data from “This study” refer to Patient 1.

## Data Availability

The data generated and analyzed in this study are included in this article and Supplementary Table S1. Additional data are available from the corresponding author on reasonable request, subject to applicable ethical and privacy restrictions.

## Supplementary Materials

The following supporting information can be downloaded at: https://www.mdpi.com/article/10.3390/genes17101260/s1, Table S1: Individual-level clinical and genetic data for reported *SLC52A2*-related RTD2 cases; Table S2: In silico predictions of the functional impact of *SLC52A2* variants identified in this study.

## Abbreviations

The following abbreviations are used in this manuscript:

AAV9	adeno-associated virus serotype 9
ABR	auditory brainstem response
ACMG	American College of Medical Genetics and Genomics
AMP	Association for Molecular Pathology
AN	auditory neuropathy
BMI	body mass index
CADD	Combined Annotation Dependent Depletion
CM	cochlear microphonic
CN	cochlear nerve
DNA	deoxyribonucleic acid
DPOAEs	distortion product otoacoustic emissions
EEG	electroencephalography
EMG	electromyography
FN	facial nerve
IVN	inferior vestibular nerve
LP	likely pathogenic
LRT	likelihood ratio test
MRI	magnetic resonance imaging
NA	not applicable
REVEL	Rare Exome Variant Ensemble Learner
RFVT2	riboflavin transporter 2
RTD	riboflavin transporter deficiency
RTD2	riboflavin transporter deficiency type 2
SARA	scale for the assessment and rating of ataxia
SIFT	Sorting Intolerant From Tolerant
SVN	superior vestibular nerve
UPR	unfolded protein response
VAS	visual analog scale
VEP	visual evoked potential
VUS	variant of uncertain significance
WES	whole exome sequencing
